# Grain Refinement and Mechanical Properties of Cu–Cr–Zr Alloys with Different Nano-Sized TiC_p_ Addition

**DOI:** 10.3390/ma10080919

**Published:** 2017-08-08

**Authors:** Dongdong Zhang, Fang Bai, Yong Wang, Jinguo Wang, Wenquan Wang

**Affiliations:** Key Laboratory of Automobile Materials of Ministry of Education, Department of Materials Science and Engineering, Jilin University, Changchun 130025, China; ddzhang14@mails.jlu.edu.cn (D.Z.); baifang14@mails.jlu.edu.cn (F.B.); wyong15@mails.jlu.edu.cn (Y.W.)

**Keywords:** microstructure evolution, nano-sized TiC_p_, heterogeneous nuclei, lattice misfit

## Abstract

The TiC_p_/Cu master alloy was prepared via thermal explosion reaction. Afterwards, the nano-sized TiC_p_/Cu master alloy was dispersed by electromagnetic stirring casting into the melting Cu–Cr–Zr alloys to fabricate the nano-sized TiC_p_-reinforced Cu–Cr–Zr composites. Results show that nano-sized TiC_p_ can effectively refine the grain size of Cu–Cr–Zr alloys. The morphologies of grain in Cu–Cr–Zr composites changed from dendritic grain to equiaxed crystal because of the addition and dispersion of nano-sized TiC_p_. The grain size decreased from 82 to 28 μm with the nano-sized TiC_p_ content. Compared with Cu–Cr–Zr alloys, the ultimate compressive strength (σ_UCS_) and yield strength (σ_0.2_) of 4 wt% TiC_p_-reinforced Cu–Cr–Zr composites increased by 6.7% and 9.4%, respectively. The wear resistance of the nano-sized TiCp-reinforced Cu–Cr–Zr composites increased with the increasing nano-sized TiCp content. The wear loss of the nano-sized TiC_p_-reinforced Cu–Cr–Zr composites decreased with the increasing TiC_p_ content under abrasive particles. The eletrical conductivity of Cu–Cr–Zr alloys, 2% and 4% nano-sized TiCp-reinforced Cu–Cr–Zr composites are 64.71% IACS, 56.77% IACS and 52.93% IACS, respectively.

## 1. Introduction

Cu-matrix composites and Cu alloys were widely used as functional and structural materials [1,2,3,4,5,6], such as electrodes for electrical-resistance welding, electric switches, lead frames, friction pieces, as well as a cooling medium of the magnetic channel, encapsulating material on account of their good wear resistance [7], excellent electrical and thermal conductivities [8,9], and good corrosion resistance [10,11] etc. Recent research indicates that the dispersion of ceramic particles in the copper matrix could play a role in pinning dislocation movement and improving the strength of the composites, in addition, alloying elements can also enhance Cu alloy [12,13,14,15]. For example, Lu et al. [15] fabricated 40–60 vol % TiC*_x_*–TiB_2_/Cu composites by thermal explosion reaction synthesis. They found that the ultimate compressive strength increased with the ceramic content and the microhardness of the 40–60 vol % TiC*_x_*–TiB_2_/Cu composites reached 339, 404 and 448 HV, respectively. However, the electrical and thermal conductivity of Cu was reduced because of the high content of ceramic particles. Zhang et al. [2] revealed that the microhardness of solid soluted Cu–Cr–Zr alloys was 181.8 HV, which was much higher than that of pure copper (68 HV), with less decreasing electrical conductivity (70.8% IACS). Nevertheless, the microhardness is much less than those of particle-reinforced Cu matrix composites. Qiu et al. [12] fabricated the 50 vol % TiC–TiB_2_/Cu composites with different Cr content. Abrasive wear results showed that the volume loss of the 50 vol % TiC-TiB_2_/Cu composites decreased with Cr content and the composites had an extremely low wear rate compared with pure Cu. Afterwards, Qiu et al. [13] studied the influence of the addition of Zr with different content on the compressive properties of TiC-TiB_2_/Cu composites; the ultimate compressive strength (σ_UCS_), yield strength (σ_0.2_) and hardness increased with Zr content. Results demonstrated that Cu could be enhanced both by ceramic particles and Cr, Zr elements.

Among the numerous reinforcing particles, titanium carbide (TiC*_x_*) is an attractive reinforcing phase [16,17,18,19,20] due to its high modulus (470 KN/mm^2^), hardness (2800 HV) and melting point (3067 °C). Combustion synthesis is one of the in-situ methods to fabricate metal matrix composites reinforced with TiC_p_ [21,22,23,24,25,26,27,28,29,30]. There are many advantages of this method. For example, its simple preparation process can result in a low cost, and the clean particle-matrix interface leads to strong interfacial bond, and its synthetic mechanism brings about the uniform distribution of particles. Moreover, combustion synthesis can synthesize TiC_p_ with various sizes, morphologies and contents in different metal matrix [21,22,23,24,25,26,27,28,29,30,31,32,33,34,35,36,37]. A number of researches showed that the decreasing size of reinforcing particles (<100 nm) had an improvement than that of micro ones in the composites [38,39,40,41]. Therefore, nano-sized TiC particles as reinforcement have attracted many researchers’ attention.

Nano-sized TiC particles could be prepared by altering the matrix content in a Cu–Ti–C system. According to our previous study [34], nano-sized TiC*_x_* could be fabricated when the Cu content was below 30 vol % in a Cu–Ti–CNTs system. However, Cu content determined the possibility of whether combustion synthesis could be self-sustaining or not. Liang et al. [42] studied the effect of adiabatic temperature (*T*_ad_) as well as Cu content on the TiC_P_ fabricated by combustion synthesis (CS) in the Cu–Ti–C system. They found that Cu content should not exceed 67.12 wt % with corresponding *T*_ad_ no less than 1800 k for the self-sustaining reaction. As for combustion synthesis reaction in a continuous heating furnace in Cu–Ti–C system, Cu content should be no less than 94.2 wt %. As a result, it can be comfirmed that TiC*_x_*/Cu composites with low content TiC*_x_* were not able to be synthesised via CS. However, high TiC*_x_* content will greatly reduce the electrical conductivity. Accordingly, a new method used to fabricate low content TiCp-reinforced Cu–Cr–Zr composites is needed. Casting is a cost-effective and efficient method of fabricating large and shaped parts [35,43]. Ex situ is the main way of fabricating particle reinforced metal matrix composites (PRMMCs) for casting method. The microstructures of the casting alloys were able to be controlled by casting and hot treatment process [3,4]. However, compared with casting method, particle shape can be controlled and size is uniform for in situ particles. Meanwhile, unlike combustion synthesis method, casting method can realize small amount of particle addition. Accordingly, the preferred method of fabricating TiCp-reinforced Cu–Cr–Zr composites should be casting combined with combustion synthesis to exert the advantages of the two methods. As is well known, most ceramic particle and Cu belong to nonwetting system, therefore, particles reinforced Cu matrix composite confront the problem of interface bonding strength. This new method of fabricating particles reinforced Cu matrix composite by combining combustion synthesis with electromagnetic stirring casting will solve the following problems: (a) this method can solve the nonwetting problem, interfacial reaction resulting in the improvement of bonding strength. (b) compared with ex situ method, the clean interface between the reinforcing phase and the matrix, leading to a strong interfacial bonding. (c) unlike the in situ method, this method is not limited by the content, it can fabricate any content as you like. Accordingly, this method will provide a new way of fabricating ceramic particle reinforced Cu matrix composite.

Pushkar Jha et al. [44] studied TiC-reinforced Cu-4 Cu-Ti-C Ni matrix composites with different particle contents fabricated by powder metallurgy. Hardness and friction as well as wear results of composites show better wear resistance than unreinforced matrix alloy. Qiu et al. [12] prepared 50 vol % (TiC–TiB_2_)/Cu composites with different Cr additions via combustion synthesis combined with hot press, abrasive wear results revealed that the abrasive wear of the (TiC–TiB_2_)/Cu composites consists of two stages. First, the Al_2_O_3_ abrasive particles penetrate into the soft copper matrix and cut it. In the second stage, the TiC and TiB_2_ ceramic particles are exposed from the copper matrix and they act as a barrier to the micro-cutting action of the abrasives. The abrasive wear resistance of the 50 vol % (TiC–TiB_2_)/Cu composites with 7 wt % Cr addition was improved by 138% compared with pure Cu. It is clear from the above that the enhancing principles of PRMMCs are the exposed ceramic playing as barriers, which enhanced the wear resistance of the composites.

In this research, nano-sized TiCp-reinforced Cu–Cr–Zr composites were prepared by the dilution of TiC_p_/Cu master alloy into Cu–Cr–Zr alloys; the TiC_p_/Cu master alloy was fabricated by thermal explosion reaction in Cu–Ti–CNTs system. The electrical conductivity and compressive properties of the TiC_p_ reinforced Cu–Cr–Zr composites were studied. Furthermore, the wear behavior and law of the TiC_p_-reinforced Cu–Cr–Zr composite was studied. This will provide some guidance for the fabrication of TiC_p_-reinforced Cu–Cr–Zr composites and its application.

## 2. Materials and Methods

Cu powder with a particle size of ~45 μm, CNTs ~15–80 μm in length and ~10–20 nm in diameter and Ti powder ~25 μm in size were used to fabricate TiC_p_/Cu master alloys via combustion synthesis. Cu, Ti and CNTs powders were weighed accurately, which was calculated according to the C/Ti mole ratio of 0.8:1. The reactants’ compositions was 70% Cu in volume. The prepared powders were milled on a planetary mixer with six cylindrical ceramic inwall containers at the speed of 50 rpm for 24 h. The blended powder was compressed into cylindrical compacts under the pressure of 100 MPa. The size of the compacts was 29 mm in diameter and 45 mm in height. Afterwards, the compacts were placed into a high strength graphite mold, which was later put into a self-made vacuum furnace and heated. W5-Re26 thermocouples were used to measure the temperature. When the temperature rose rapidly, stopped heating and pressed with the pressure of 40 MPa. The nano-sized TiC_p_/Cu master alloy was obtained when it was cooled down to room temperature.

The Cu–Cr–Zr composites reinforced with different TiC_p_ content were prepared by the dilution of TiC_p_/Cu master alloy into Cu–Cr–Zr alloys via stirring casting. At first, the prepared Cu–Cr–Zr alloys were heated in a high purity graphite crucible, which was later placed in an induction furnace with a water cooling system. Once the Cu–Cr–Zr alloys melted, the TiC_p_/Cu master alloy was added in the molten Cu–Cr–Zr. Manual stirring was helpful to melt and disperse the master alloy. Once the master alloy melted completely, kept the temperature for 3 min and the melt were poured into a steel mold. After cooling down to indoor temperature, the TiCp-reinforced Cu–Cr–Zr composites with different TiC_p_ content were successfully prepared.

The characterization of phase constitutions of TiC_p_/Cu master alloy were performed on a X-ray diffraction (XRD, Rigaku D/Max 2500PC, Tokyo, Japan) with Cu Kα radiation at the scanning speed of 4°/min. The TiC_p_ in TiC_p_/Cu master alloy were extracted by FeCl_3_-HCl distilled water solution, the morphologies of TiC_p_ were detected by Field Emission Scanning Electron Microscope (FESEM, JSM 6700, Tokyo, Japan). Olympus optical microscope (XJZ-6, Tokyo, Japan) and high resolution transmission electron microscopy (HRTEM, JEM-2100F, Tokyo, Japan) were used to observe the microstructure of the Cu–Cr–Zr alloys and TiCp-reinforced Cu–Cr–Zr composites. The compressive properties were proceeded on a servo hydraulic materials testing system (MTS, MTS 810, Minneapolis, MN, USA) at a strain rate of 3 × 10^−4^ s^−1^. Microhardness of the Cu–Cr–Zr alloys and TiCp-reinforced Cu–Cr–Zr composites were performed by a Vickers hardness tester (Model 1600-5122VD, Newage, Feasterville, PA, USA) using a static load of 50 gf and a dwell time of 15 s. Abrasive wear tests were carried out on a pin-on-disk machine with the SiC abrasive papers, the load was 5 N and the wear distance was 24.78 m. The abrasive wear samples are 12 mm in height and 6 mm in diameter. The electrical conductivities of the Cu–Cr–Zr alloys and TiCp-reinforced Cu–Cr–Zr composites were measured at room temperature by a digital eddy current electroconductive machine (Sigma 2008b, Xiamen, China), International Annealed Copper Standard (IACS) are quoted as the units of electrical conductivity results.

## 3. Results and Discussion

**Figure 1:**
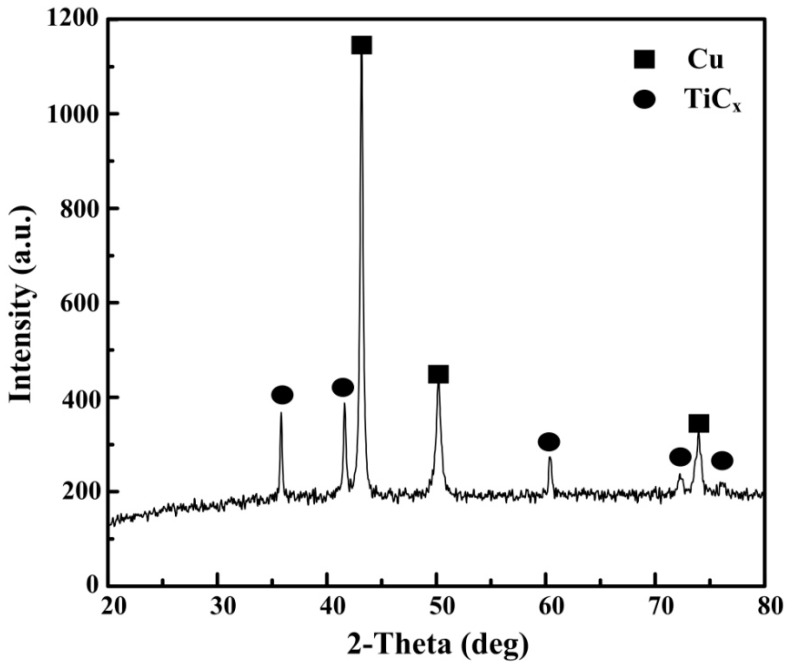
XRD patterns of Cu–Ti–CNTs master alloy after combustion synthesis.

**Figure 2:**
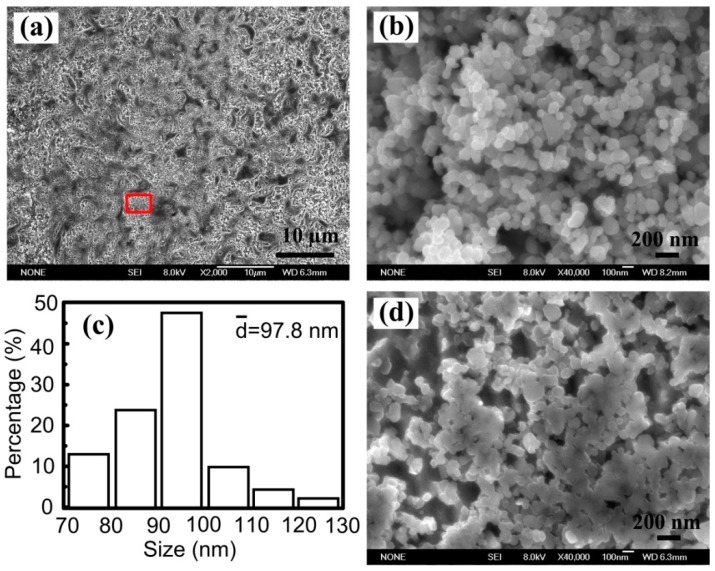
(**a**) Morphology of the deep etched surfaces of the master alloy, (**b**) the TiC_p_ extracted from the TiC_p_/Cu master alloy, (**c**) size distribution of extracted TiC_p_ and (**d**) enlarged image corresponding to selected area.

Figure 1 shows the XRD results of Cu–Ti–CNTs master alloy fabricated by combustion synthesis. The diffraction peaks belonging to TiC_p_ and Cu can be seen clearly, indicating that the in-situ nano-sized TiC_p_/Cu master alloy was successfully fabricated. Figure 2a shows the morphology of the deep etched surfaces of the nano-sized TiC_p_/Cu master alloy. As can be seen, Cu was removed and the TiC_p_ in the TiC_p_/Cu master alloy exhibited a homogeneous distribution. The morphologies of extracted nano-sized TiC_p_ were shown in Figure 2b. As indicated, the morphologies of the extracted TiC_p_ in TiC_p_/Cu master alloy are spherical, with the size of 100 nm on average (Figure 2c). The interspace between TiC_p_ of the magnified image of the selected area can be observed (Figure 2d).

**Figure 3:**
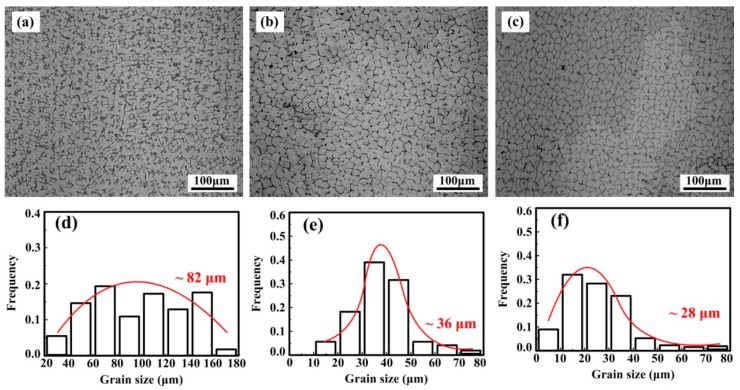
The morphologies of grain in (**a**) Cu–Cr–Zr alloys, (**b**) 2-wt %, (**c**) 4-wt % nano-sized TiCp-reinforced Cu–Cr–Zr composites and (**d**–**f**) the corresponding grain size distribution.

The microstructures of nano-sized TiC_p_/Cu–Cr–Zr composites with different TiC_p_ content are shown in Figure 3. As illustrated, dendritic grain is the main morphology of Cu–Cr–Zr alloys with the grain size of 82 μm on average; the morphologies of the grain in 2 wt % and 4 wt % nano-sized TiCp-reinforced Cu–Cr–Zr composites are equiaxed crystal and the grain size are 36 μm and 28 μm on average, respectively. The morphology of the grain changes from coarse dendrites to equiaxed crystal with increasing TiC_p_ content. An investigation [45] suggested that TiC_p_ were not the effective substrates for the heterogeneous nucleation of the Cu-melt. However, the variation in grain from dendrites to equiaxed crystal was a piece of adverse evidence for the ineffectiveness of TiC_p_ in grain refinement in Cu alloy.

**Figure 4:**
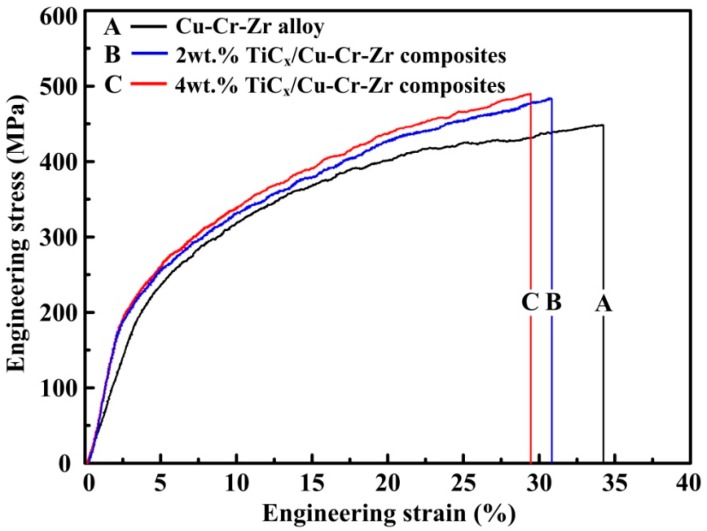
Engineering stress-strain curves of compressive test for (**A**) Cu–Cr–Zr alloys, (**B**) 2-wt % and (**C**) 4-wt % nano-sized TiCp-reinforced Cu–Cr–Zr composites

Figure 4 shows the engineering stress-strain curves of compressive test for Cu–Cr–Zr alloys and nano-sized TiCp-reinforced Cu–Cr–Zr composites with different TiC_p_ content. The compressive test data are presented in Table 1. As exhibited in the diagram, the yield strength (σ_0.2_), ultimate compressive strength (σ_UCS_) and microhardness (HV) of Cu–Cr–Zr alloys were obviously enhanced at the expense of the fracture strain (ε_f_) because of the addition of TiC_p_. For the nano-sized TiCp-reinforced Cu–Cr–Zr composites, the σ_0.2_, σ_UCS_ and microhardness increased with the increasing TiC_p_ content. The 4-wt% TiCp-reinforced Cu–Cr–Zr composites possessed the highest yield strength (σ_0.2_), ultimate compressive strength (σ_UCS_) and microhardness (HV), which are 190 MPa, 491 MPa and 118.5 HV, respectively. The fracture strain (ε_f_) of Cu–Cr–Zr alloys, 2-wt % and 4-wt % nano-sized TiCp-reinforced Cu–Cr–Zr composites are 34.1%, 30.7% and 29.3%, respectively.

**Figure 5:**
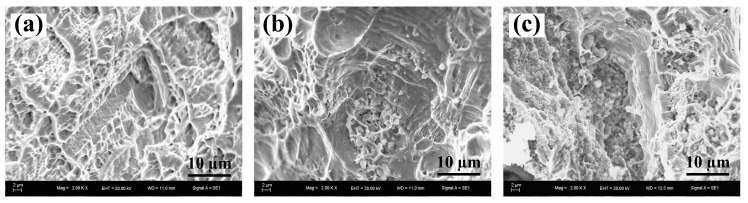
The fracture surface of (**a**) Cu–Cr–Zr alloys, (**b**) 2-wt % nano-sized TiC_p_/Cu–Cr–Zr composites and (**c**) 4-wt % nano-sized TiC_p_/Cu–Cr–Zr composites.

As we know, the metal matrix determined the ductility of the composites and the ceramic particles determined the strength of the composites [46]. The fracture surface of (a) Cu–Cr–Zr alloys, (b) 2-wt % nano-sized TiC_p_/Cu–Cr–Zr composites and (c) 4-wt % nano-sized TiC_p_/Cu–Cr–Zr composites were shown in Figure 5. As can be seen, the fracture surface of Cu–Cr–Zr alloys consists of dimples, which means that the Cu–Cr–Zr alloy has a good plasticity. With the addition of TiC_p_, the amount of dimples decreased and some TiCp clusters appeared in the dimples. Uniformly dispersed TiC_p_ can improve the mechanical properties of the Cu–Cr–Zr alloys, but cluster of TiC_p_ will act as the source of crack leading to the reduction of the plasticity. This means that the additon of TiC_p_ can improve the strength of composite but sacrifice the plasticity. According to the above viewpoint, we conject that the σ_0.2_, σ_UCS_ and microhardness could be enhanced and the fracture strain (ε_f_) decreased with the increasing TiC_p_ content. Consequently, the 4-wt % TiCp-reinforced Cu–Cr–Zr composites possessed the highest compression strength and microhardness (HV).

**Figure 6:**
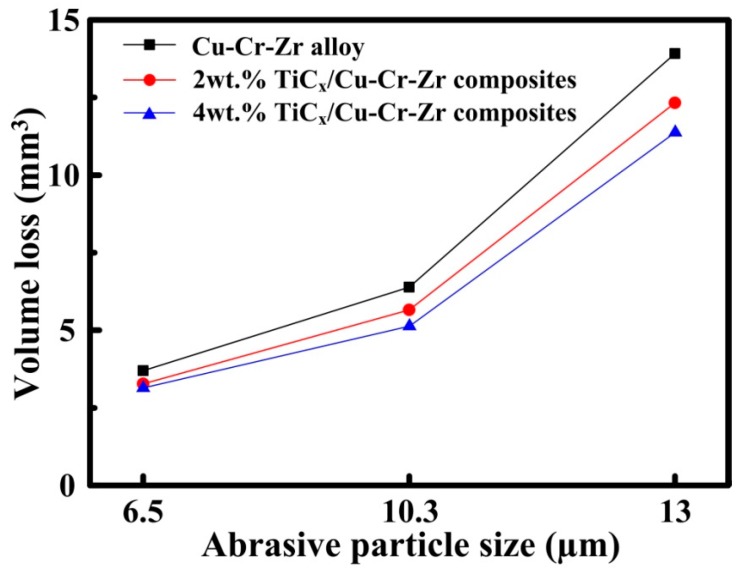
Volume loss against different abrasive particles of 6.5 μm, 10.3 μm and 13 μm at the applied load of 5N for the Cu–Cr–Zr alloys and nano-sized TiCp-reinforced Cu–Cr–Zr composites.

**Figure 7:**
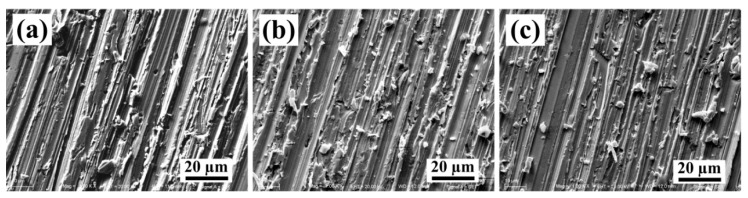
The morphologies of the worn surfaces for (**a**) Cu–Cr–Zr alloys, (**b**) 2-wt % and (**c**) 4-wt % nano-sized TiCp-reinforced Cu–Cr–Zr comp osites at 13 μm abrasive particles.

The variation in volume loss with different abrasive particles of 6.5 μm, 10.3 μm and 13 μm at the applied load of 5 N for the Cu–Cr–Zr alloys, 2-wt % and 4-wt % nano-sized TiCp-reinforced Cu–Cr–Zr composites are shown in Figure 6. As indicated, the volume loss of the alloys and composites increased with the increasing abrasive particles. This may be due to the large abrasive particles that penetrated deeply into contact area of the alloys and the composites, leading to the increase in volume loss of composites. Meanwhile, the volume loss of the Cu–Cr–Zr alloys, 2-wt % and 4-wt % TiCp-reinforced Cu–Cr–Zr composites decreased with the increasing TiC_p_ content under all abrasive particles. The reason may be the grain refinement, nano-sized particle strengthening and the good interface bonding of composites, which could enhance the hardness and decrease the volume loss of composites. It also can be confirmed by the SEM images of the worn surfaces for Cu–Cr–Zr alloys, 2-wt % and 4-wt % nano-sized TiCp-reinforced Cu–Cr–Zr composites at 13 μm abrasive particles, as shown in Figure 7.

SiC particles penetrated deeply into the contact area of Cu–Cr–Zr alloys and these abrasive particles (Figure 7a) lead to the deformation of the surfaces of the alloys. The surfaces, after wear and tear of TiCp-reinforced Cu–Cr–Zr composites, became smooth with the increasing nano-sized TiC_p_ contents (Figure 7b,c). As already discussed, microhardness of the TiCp-reinforced Cu–Cr–Zr composites can be improved with an increase in particle content. Therefore, the depth of abrasive particles penetrated into the composites decreased with the increasing hardness. Accordingly, it is evident that TiC_p_ can effectively enhance the microhardness and wear resistance by dispersing TiC_p_ into Cu matrix, in which TiC_p_ played a role as grain refinement in enhancing the composites and a barrier in reducing the cutting efficiency of abrasive particles as well as hammering the deformation of the Cu matrix. Accordingly, the wear resistance of the TiCp-reinforced Cu–Cr–Zr composites was enhanced due to the addition of TiC_p_.

**Figure 8:**
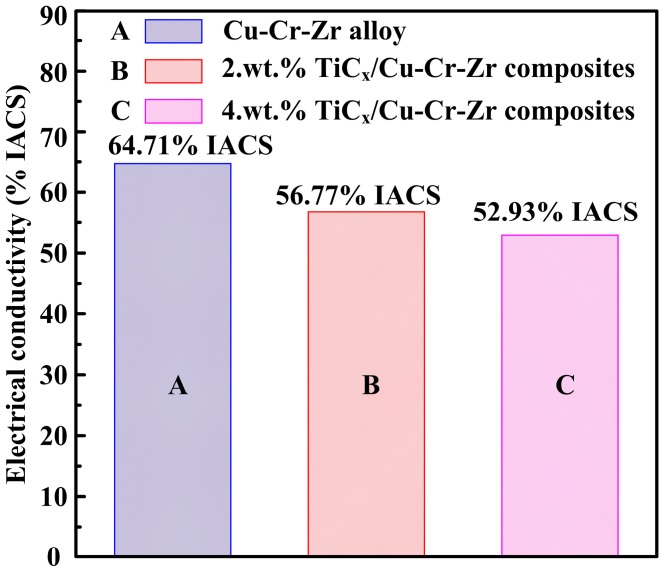
The electrical conductivity of Cu–Cr–Zr alloys and TiCp-reinforced Cu–Cr–Zr composites.

Figure 8 shows the electrical conductivity of Cu–Cr–Zr alloys and nano-sized TiCp-reinforced Cu–Cr–Zr composites with different TiC_p_ content. As indicated, their electrical conductivities are 64.71%, 56.77% and 52.93% IACS, respectively. The electrical conductivities decrease with the addition of TiC_p_, which are in the order of Cu–Cr–Zr alloys > 2-wt % TiCp-reinforced Cu–Cr–Zr composites > 4-wt % TiCp-reinforced Cu–Cr–Zr composites.

**Figure 9:**
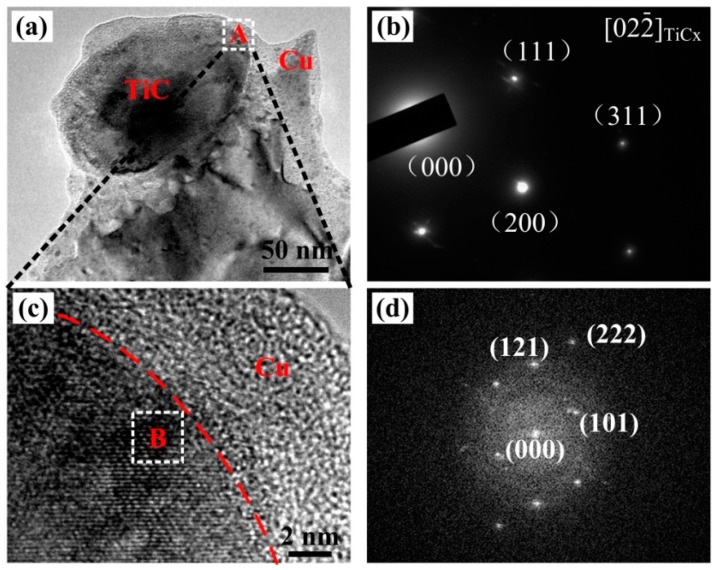
(**a**) TEM images of 4 wt % nano-sized TiCp-reinforced Cu–Cr–Zr composites, (**b**) the images of TiC_p_ electron diffraction pattern, (**c**) the HRTEM image corresponding to area A, and (**d**) the FFT images corresponding to area B.

Wetting angle (θ) was a standard to measure whether the reinforced particles can be act as the heterogeneous nuclei to refine the grain during the crystallization of Cu melt. Small wetting angle (0° < θ < 90°) can provide the potential to act as the heterogeneous nuclei and high wetting angle (90° < θ < 180°) can not. TiC_p_ and Cu have a high wetting angle that belongs to non-wetting system. Accordingly, the TiC_p_ could not act as the heterogeneous nuclei to refine the grain of Cu–Cr–Zr alloy. For the purpose of studying the grain refining mechanism of Cu–Cr–Zr alloys, we therefore studied the interfaces between TiC_p_ and Cu nmatrix in the TiCp-reinforced Cu–Cr–Zr composites. Figure 9 shows TEM images of 4 wt % nano-sized TiCp-reinforced Cu–Cr–Zr composites. As indicated in Figure 9a, nano-sized TiC_p_ can be seen clearly in the Cu composites. The electron diffraction pattern image of marked area in Figure 9b corresponds to the [02$\overset{-}{2}$] zone axis on TiC_p_ in Figure 9a. The HRTEM image (Figure 9c) of area A in Figure 9a and the fast-Fourier-filtered (FFT) images (Figure 9d) of selected area in Figure 9c are consistent with the [10$\overset{-}{1}$] zone axis of Cu_3_Ti. The lattice vectors (a, b, and c) of space group (Pmmn) belonging to Cu_3_Ti (Pmmn, *a* = 5.162 nm, *b* = 4.347 nm, *c* = 4.531 nm) can be calculated and confirmed around the TiC_p_ according to the HRTEM and FFT. A layer of Cu_3_Ti existed between the TiC_p_ and the Cu–Cr–Zr matrix and a good interfacial bonding in the HRTEM were presented between nano-sized TiC_p_ and Cu–Cr–Zr matrix. Fan et al. [47] confirmed that the free Ti in the melt was conducive to the formation of Cu_3_Ti layer. Moreover, Yang et al. [48] indicated that the wear resistance could be improved for the good interface bonding of reinforced particles and the matrix in the composites.

Lattice misfit (*δ*) used to judge the possibility of the heterogeneous nuclei can be calculated by the flowing mathematical model [49]:

(1) $$\delta_{{\left(\mathrm{hkl}\right)}_{n}}^{{\left(\mathrm{hkl}\right)}_{s}}=\frac{1}{3}\sum_{i=1}^{3}\frac{\left|d{\left[\mathrm{uvw}\right]}_{s}^{i}\cos\theta -d{\left[\mathrm{uvw}\right]}_{n}^{i}\right|}{d{\left[\mathrm{uvw}\right]}_{n}^{i}}\times 100\%$$

where the (hkl)_s_ and (hkl)_n_ are the low index crystal face of the nucleus and the matrix; [uvw]_s_ and [uvw]_n_ are the low index crystal orientation in the (hkl)_s_ and (hkl)_n_; *d*[uvw]_s_ and *d*[uvw]_n_ are the atom space along the direction of the [uvw]_s_ and [uvw]_n_; θ is the angle between [uvw]_s_ and [uvw]_n_.

**Figure 10:**
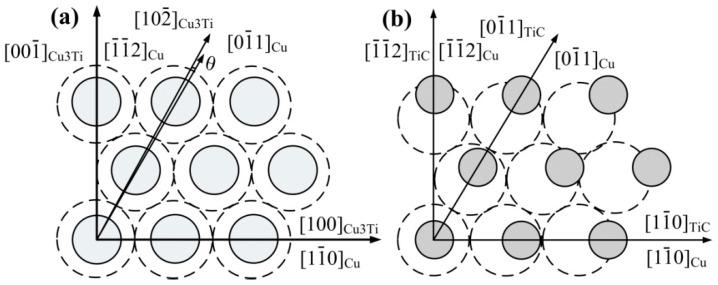
Crystallographic relationship. (**a**) (010) Cu_3_Ti and (111) Cu faces, (**b**) (111)_TiC_ and (111)_Cu_ faces.

The crystallographic relationships for Cu_3_Ti and Cu as well as TiCp and Cu are shown in Figure 10. The (111) of Cu is superimposed on the (010) of Cu_3_Ti, as shown in Figure 10a. The low index crystal faces of Cu are [1$\overset{-}{1}$0], [0$\overset{-}{1}$1] and [$\overset{-}{1}$$\overset{-}{1}$2], and that of Cu_3_Ti are [100], [10$\overset{-}{2}$] and [00$\overset{-}{1}$]. The angle between [0$\overset{-}{1}$1] of Cu and [10$\overset{-}{2}$] of Cu_3_Ti is 0.33°. The low index crystal faces of Cu_3_Ti are [010], [01$\overset{-}{1}$] and [00$\overset{-}{1}$], and that of TiC are [001], [011] and [00$\overset{-}{1}$]. The angles are 0°, 0.7884° and 0°, respectively.

Table 2 shows the calculated results of lattice misfit using Equation (1). As shown in Table 2, the lattice misfit (δ) between the (111) of Cu and the (010) of Cu_3_Ti is 1.9%, indicating that Cu_3_Ti could act as the heterogeneous nuclei during the crystallization of Cu-melt. On the other hand, the lattice misfit (δ) between the (100) of Cu_3_Ti and the (100) of TiC is 1.2%. The low lattice misfit (δ) between Cu_3_Ti and Cu as well as Cu_3_Ti and TiCp is conducive to good interface bonding. According to the reaction mechanism in Cu–Ti–CNTs system [25], Cu firstly reacts with Ti to form Ti_x_Cu_y_ around Ti particles. With increasing temperature, Ti_x_Cu_y_ melts to form a Cu–Ti binary liquid phase, meanwhile, CNTs dissolve in the Cu–Ti binary liquid phase to form an Cu–Ti–C ternary liquid phase. When the concentration of [Ti] and [C] in the Cu–Ti–C ternary liquid phase is high enough for reactions between [Ti] and [C] to occur, TiC*_x_* will be synthesized and precipitate out of the melts. However, the reaction is not complete; Ti*_x_*Cu*_y_* will be remaining in the TiC*_x_*/Cu master alloy. When the master alloy remelts and is dispersed into the melting Cu–Cr–Zr alloys, the Cu_3_Ti layer formed by the adsorption of Ti atoms from Cu–Ti solution, and the Cu_3_Ti could even exist at lower concentration or at the temperature above the alloy liquidus. Therefore, the formation of Cu_3_Ti is mainly because of the reduction of the interfacial energy at the interface between TiCp and the Cu-melt. As a result, Cu_3_Ti formed surrounding the TiCp as the heterogeneous nuclei refined the grain.

As already discussed, the grain refinement of Cu–Cr–Zr alloy is caused by the Cu_3_Ti, which formed on the surface of TiCp. TiCp with a Cu_3_Ti layer could be used as the heterogeneous nuclei of the Cu-melt. The refinement of grain, nano-sized particle strengthening and good interface bonding improved the ultimate compressive strength (σ_UCS_), yield strength (σ_0.2_), microhardness (HV) and wear resistance of different TiCp-content reinforced Cu–Cr–Zr composites with a slight decrease in fracture strain (ε_f_) and electrical conductivity.

## 4. Conclusions

The compressive properties, wear resistance and electrical conductivities of Cu–Cr–Zr alloys and TiCp-reinforced Cu–Cr–Zr composites fabricated via thermal explosion combined with stirring casting were investigated. The results show that nano-sized TiC_p_ can effectively refine the grain of Cu–Cr–Zr alloys. Nano-sized TiC_p_ was surrounded by a layer of Cu_3_Ti, which played a role as heterogeneous nuclei of Cu. The microstructure of Cu–Cr–Zr composites changed from dendritic grain to equiaxed crystal with the addition of TiC_p_/Cu master alloy. The grain size decreased from 82 to 28 μm with the increasing TiC_p_ content. Compared with Cu–Cr–Zr alloys, the ultimate compressive strength (σ_UCS_), yield strength (σ_0.2_) and hardness (HV) of nano-sized TiCp-reinforced Cu–Cr–Zr composites were improved. The wear resistance of Cu–Cr–Zr alloys and TiCp-reinforced Cu–Cr–Zr composites increased with the increasing content of TiCp. The refinement of grain, nano-sized particle strengthening and the good interface bonding improved the strength, hardness and wear resistance of Cu–Cr–Zr alloys and nano-sized TiCp-reinforced Cu–Cr–Zr composites with different particle content.

## Figures and Tables

**Table 1:** Compressive properties of the Cu–Cr–Zr alloys, 2-wt % and 4-wt % TiCp-reinforced Cu–Cr–Zr composites.

Samples	σ_0.2_/MPa	σ_UCS_/MPa	ε_f_/%	Hardness (HV)
Cu–Cr–Zr alloys	$178_{-14}^{+12}$	$449_{-13}^{+10}$	$34.1_{-0.5}^{+0.2}$	97.1
2-wt % TiC_p_/Cu–Cr–Zr	$187_{-13}^{+9}$	$484_{-11}^{+12}$	$30.7_{-0.5}^{+0.2}$	112.3
4-wt % TiC_p_/Cu–Cr–Zr	$190_{-11}^{+8}$	$491_{-9}^{+12}$	$29.3_{-0.2}^{+0.3}$	118.5

**Table 2:** The calculated lattice misfit between Cu_3_Ti and α-Cu phases.

Matching Plane	[uvw]_Cu3Ti_	[uvw]_Cu_	θ (deg)	δ (%)
(010)_Cu3Ti_//(111)_Cu_	[100]_Cu3Ti_	[1$\bar{1}0$]_Cu_	0	1.9
	[1$0\bar{2}$]_Cu3Ti_	[$0\bar{1}$1]_Cu_	0.33	
	[00$\bar{1}$]_Cu3Ti_	[$\bar{1}\bar{1}2$]_Cu_	0	
(111)_TiC_//(111)_Cu_	[1$\bar{1}$0]_TiC_	[1$\bar{1}0$]_Cu_	0	19.8
	[0$\bar{1}$1]_TiC_	[0$\bar{1}$1]_Cu_	0	
	[$\bar{1}\bar{1}$2]_TiC_	[$\bar{1}\bar{1}$2]_Cu_	0	
(100)_Cu3Ti_//(100)_TiC_	[010]_Cu3Ti_	[001]_Ti__C_	0	1.2
	[01$\bar{1}$]_Cu3Ti_	[011]_Ti__C_	0.7884	
	[00$\bar{1}$]_Cu3Ti_	[010]_TiC_	0	
